# A high-speed current source for magnetorheological applications

**DOI:** 10.1038/s41598-023-43730-6

**Published:** 2023-10-16

**Authors:** Lei Xie, Chuan Lu, Jianfei Yin, Bo Wei, Yuhao Wang, Pengsai Wang, Zhipeng Yang, Changrong Liao

**Affiliations:** 1 Key Laboratory of Optoelectronic Technology and Systems (Education Ministry of China), Chongqing University, Chongqing, 400044 People’s Republic of China; 2 Science and Technology on Reactor System Design Technology Laboratory, Nuclear Power Institute of China, Chengdu, Sichuan Province, 610213 People’s Republic of China; 3 Laboratory of Science and Technology on Integrated Logistics Support, National University of Defense Technology, Changsha, Hunan Province, 410073 People’s Republic of China

**Keywords:** Mechanical engineering, Techniques and instrumentation

## Abstract

Current source is an indispensable component of magnetorheological (MR) systems. Though MR fluid has a phase change as fast as in 1 ms, the response of MR damper (MRD) to generate the damping force may be two orders of magnitude longer. Therefore, the rapid response of current source is a key to realize the real-time semi-active control of MR devices. This study proposes a programmable high-speed, low-cost current source exclusively for MR devices based on the synergy between supercapacitor and Buck converter (i.e., SSBC current source). SSBC current source features a strategy consisting of a lifting phase of supercapacitor and a following maintaining phase of Buck converter. Specifically, the high power density of supercapacitor contributes to rapidly lifting/raising the initial current, and then, like a “relay race”, the expected output is maintained through a Buck converter. Theoretical modeling and experiments are performed systematically. The response times (@ 95% of expected outputs) measured are 0.44, 0.84 and 1.88 ms for the outputs of 3, 6 and 9 A, respectively; these values are highlighted as the fastest level in this field. Besides, the response can be up to 24.6 and 43.7 times faster than the cases using supercapacitor and Buck converter to directly drive the MRD, respectively. SSBC current source is employed to generate a sequence of currents/magnetic inductions, only four variables of which need to be controlled programmatically: the order of lifting and maintaining phases, switching time of lifting phase, PWM duty cycle of Buck converter and duration of maintaining phase. The response time stability is verified by 100 cycles of on/off tests, showing a fluctuation of only 1.1%, which indicates a very reliable high-speed response. This study provides an exclusive power supply with a novel strategy for MR devices, which is believed to be an important promotion for MR technologies.

## Introduction

Magnetorheological (MR) fluid is an intelligent material containing micron-sized soft magnetic particles, non-magnetic carrier fluid and functional additives. The unique MR effect of MR fluid lies in the reversible phase change between liquid and solid under the control of an external magnetic field. This phase change happens extremely fast and typically takes around 1 ms^1,2^, with a significant change of up to 100 times in yield stress, viscosity and other rheological properties. Such benefits have enabled MR fluid and derived MR devices to become a prior technology in the development of semi-active vibration and shock controls, such as MR dampers (MRDs hereinafter) in vehicle suspensions, landing gears, and anti-recoil systems^3–6^. In addition, there are extensive applications in intelligent clutches, optical glass polishing and other fields^7,8^. The control of MR devices is realized by regulating the strength of magnetic field in the channel which MR fluid flows through. In this case, it is actually to control the current of the excitation coil that produces the magnetic field. Then, the rheology of MR fluid is controlled and, hence, the performance of the MR devices (e.g., damping force of MRD) is controlled in real time.

MRD is a typical example of MR devices. As a semi-active actuator, the response time of MRD is a key parameter that determines its real-time controllability. Especially in impact cases, the operation process is extremely short (several to tens of ms). In order to obtain a desired damping effect, MRD is expected to respond instantaneously to absorb the vibrations and impacts. More practically, the MR fluid is to be regulated by a sequence of currents, rather than a constant current, to optimally produce a sequence of damping forces. However, although the adjustment of MR fluid is performed as fast as within 1 ms, the total response time of MRD to produce the final damping force may take up to two orders of magnitude longer. For example, a study showed that the response time of the MRD was up to 90 ms^9^. The primary reasons are the high inductance of the excitation coil and the eddy current of the iron core material^10,11^. The magnetic field in the damping channel of MRD is generated by the excitation coil (with hundreds of windings), whose high inductance plays an important role in preventing the rapid response of current^12^. Some researchers have focused on how to reduce the inductance of coil, such as using multiple coils in parallel rather than in series^10^. Its actual effect is not significant, but the current intensity becomes doubled and redoubled so as to achieve the same magnetic field strength. Strecker et al.^13^ were committed to reducing the iron core eddy current by using a grooved configuration to speed up the response of the MRD to 1.6 ms. On the other hand, this grooved design degraded the magnetic field, resulting in a decrease in damping force. Thus, it may not be suitable for applications where large damping force is required. Overall, it can be seen that rapid response of MRD is a key to realize its real-time control. In this study, we directly propose an exclusive high-speed current source for MR devices, instead of focusing on their response time related factors.

Low-cost current source is an indispensable component in MR systems for their affordable commercial applications. Researchers have carried out studies on current sources for MRD. Yang et al.^14^ presented that current source has a faster response than voltage source. Zheng and Zhang et al.^9,15^ investigated the equivalent circuit of the MRD and designed an advanced correcting circuit to shorten the response time. They obtained a response time of 5 ~ 65 ms for a current output of 0 ~ 2 A. Their experiment also showed that a PID controller could accelerate the response. Buck converter, as a common non-isolated step-down DC-DC topology, features a simple and reliable structure. Pulse width modulation (PWM) wave is usually used to regulate its output. Tong et al.^16^ established a Buck converter model in peak current mode, and analyzed its dynamic process with an MRD as the load. Liu et al.^17^ designed a Buck current source for an automotive MRD, with an output of 0 ~ 2 A and a response time of around 5 ms. Strecker et al.^18^ realized a current source by applying an overvoltage input. Their output range was 0 ~ 2 A and a 2 A current could be generated in 0.23 ms. To achieve this fast response, the authors also used ferrite for the piston head to reduce the eddy current effect. As mentioned in above reports, we know that the output range of current sources for MRDs are mostly 0 ~ 2 A.

Supercapacitor is a promising energy storage element featuring high power density, fast charge and discharge as well as long life. Supercapacitor battery technology has received particular attentions in recent years, for instance, in electromagnetic catapults, rail transportation, and electric vehicles^19–23^. Regarding MRD, it is required to respond quickly to stimulus of vibrations and shocks. Essentially, it is the value of excitation current that must change rapidly. Besides, the intensity of current should be large, typically several Amperes. We find that the features of supercapacitor meet exactly these requirements. In addition, Buck converter possesses advantages in terms of continuous adjustability, high accuracy, small ripple, and simple and reliable structure^24–26^. Another feature is that the output of Buck converter is proportional to the duty cycle of the PWM signal. Therefore, a combination of supercapacitor with Buck converter could be an economical strategy to achieve fast discharge and high current output for MRD. Further, a current sequence regulated by the PWM of Buck converter can be realized to satisfy the real operating conditions of MRD.

Inspired by these, this study proposes a programmable high-speed current source for MR devices based on synergy between supercapacitor and Buck converter (SSBC for short). We denote the current source as SSBC current source hereinafter. First, the design strategy of the SSBC current source and the equivalent circuit model with an MRD as the load are presented. Second, theoretical modelling is carried out for circuits incorporating supercapacitor directly as a current source (SD current source hereinafter) for MRD and Buck converter directly as a current source (BCD current source hereinafter) for MRD, respectively. Third, we obtain the simulated responses of SD, BCD and SSBC current sources, along with the related response of magnetic field in MRD. Then, results from experiments are given and discussed to compare with the simulated ones, as well as to verify the superiority of the SSBC current source. At last, the stability of response time of the SSBC current source is presented by 100 cycles of on/off repeatability tests.

## Design strategy of the SSBC current source

### Equivalent circuit with MRD as a load

**Figure 1:**
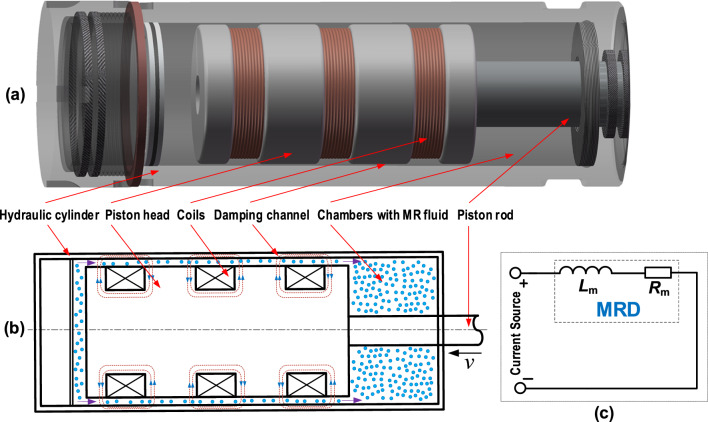
(**a**) 3D^27^ and (**b**) 2D schematics of an MRD, and (**c**) a simple equivalent circuit with the MRD (i.e., an inductive coil) as a load.

The structure of an MRD is mainly composed of piston rod, piston head, hydraulic cylinder, chambers and excitation coil, as shown in Fig. 1a and b^27^. The three-stage excitation coil is the only electrical component. The piston head, driven by external vibrations and shocks, pushes MR fluid to flow through the channel to produce an adjustable damping force. The coil generates a magnetic field in the channel when a current is applied by a current source. By regulating the value of output current of the current source, the value of damping force of MRD is adjusted to realize its semi-active control. As an electrical component constituted by an equivalent inductor *L*_m_ and a equivalent resistor *R*_m_, the coil works as a load of the current source (see Fig. 1c). There is an induced current generated by the coil, which is against the varying trend of the applied current. When the applied current increases, it is hindered by the induced current generated in the converse direction. When the applied current decreases, the induced current generated in the same direction struggles to help it to sustain for a longer time. The eddy current in the iron core plays a similar role in the response of magnetic field. In order to realize a fast control of MRD, we need to apply expected values of current to the coil as rapidly as possible. Therefore, one challenge of designing the current source for MRD is to overcome the effect of the induced current of inductive coil as well as the eddy current.

In Fig. 1c, the equivalent inductor *L*_m_ has a series connection with the resistor *R*_m_. Simply, the current *i*(*t*) in the circuit as a function of time *t* is

1 $$i(t) = \frac{U}{{R_{{\text{m}}} }}\left( {{\text{l}} - {\text{e}}^{{ - \frac{t}{\tau }}} } \right)$$

where *U* is the voltage applied to the coil, and *τ* = *L*_m_ / *R*_m_ is the time constant of the first-order system. To speed up the response, i.e., to reduce the time constant *τ*, we can decrease* L*_m_ or increase *R*_m_. However, they are usually limited to actual conditions for a given MRD. Here, we focus on the current source itself rather than the factors in MRD.

### Design strategy

**Figure 2:**
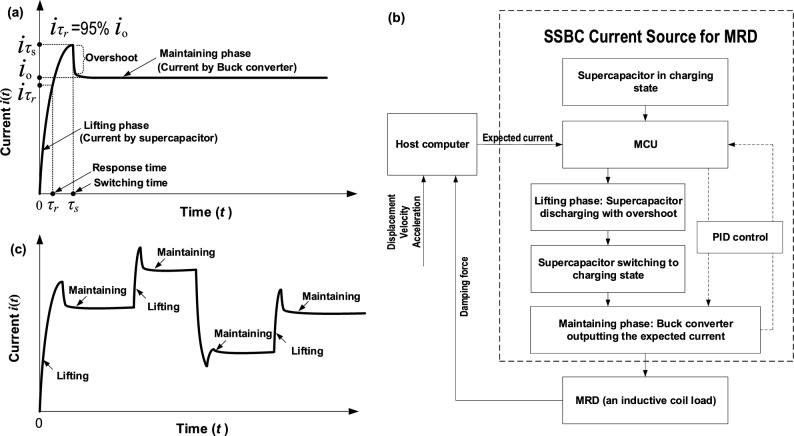
Schematic of SSBC current source (synergistic strategy with supercapacitor and Buck converter). (**a**) Realization of single value output of expected current which includes a lifting phase contributed by supercapacitor, followed by a maintaining phase contributed by Buck converter. Here the response time *τ*_*r*_ is defined as the time when the current reaches 95% of the expected output current. (**b**) Block diagram of the design strategy of SSBC current source. (**c**) Realization of output of a sequence of expected currents where the process is a repetition of the strategy for the single output shown in (**a**). The overshoots in (**a**) and (**c**) help to speed up and thus obtain a step output of magnetic induction.

Although supercapacitor has the ability of fast charge and discharge as well as large current, it cannot maintain a long-term output due to its low energy density. In contrast, it is convenient to regulate the output current of Buck converter by means of PWM, but its response is not as fast as that of supercapacitor. Therefore, we propose the SSBC current source with a synergistic strategy that combines supercapacitor with Buck converter, which is able to take advantage of both elements and avoid their disadvantages. In general, the high power density of supercapacitor with a proper initial voltage will contribute to rapidly lifting or raising the current in the initial phase (see Fig. 2a, 0 < *t* < *τ*_*s*_). Then, like a “relay race”, the expected output current is maintained through a Buck converter (Fig. 2a, *t* > *τ*_*s*_). The switching time, *τ*_*s*_, is the moment of switching from the supercapacitor to the Buck converter. We refer to these two stages as the “lifting phase” of supercapacitor and the “maintaining phase” of Buck converter, respectively. There is an intentional overshoot existing in the end of the lifting phase, which helps to speed up and thus obtain a step output of magnetic induction. Specifically, the strategy of SSBC current source is described as follows. First, it is well known that the higher the initial voltage of supercapacitor is, the faster the discharge will be, so the supercapacitor is initially charged with an appropriate high voltage. When receiving an external trigger signal, the microprocessor (MCU) switches on the supercapacitor (see Fig. 2a, 0 < *t* < *τ*_*s*_). The supercapacitor is cut off at the switching time when the current exceeds the expected value to a certain extent (i.e., the overshoot, see Fig. 2a, *t* = *τ*_*s*_). The maintaining phase follows the lifting phase. Namely, at the switching time, the MCU switches on the Buck converter to output the expected current by the adjustment of duty cycle of PWM signal (Fig. 2a, *t* > *τ*_*s*_). During the maintaining phase, the supercapacitor can be recharged to recover the lost power in preparation for the next lifting phase cycle. Figure 2b presents the overall design strategy.

The above strategy only realizes the output of a single constant current. In practical applications, a more challenging fact is that MRD should vary the damping force quickly according to environmental requirements. In other words, the SSBC current source must also change the output current rapidly, i.e., to output a sequence of currents. As illustrated in Fig. 2c, we divide this object into two aspects: the lifting phase of supercapacitor and the maintaining phase of Buck converter. For the first aspect, there is a fact that the supercapacitor merely works in the lifting phase to rapidly lift the current, but the real output current is provided by the Buck converter. We know that the supercapacitor has different output current levels ​​under different initial voltages. A higher voltage allows it to achieve a faster discharge rate. Therefore, when the quantity of electric charges of a given supercapacitor is sufficient to cover the needs of the entire current range, an initial voltage as high as possible would enable all expected currents to achieve their fastest speeds. In other words, a simple solution is to employ a maximum voltage below its safety limit (avoiding breakdown). Thus, a particular lifting phase is obtained by controlling the switching time to reach the expected current value with an overshoot. For the second aspect, the maintaining phase of Buck converter needs to maintain the output current at the desired value. This can be readily obtained by adjusting the duty cycle of PWM signal. In applications, factors such as the heating of coil will affect the output current, so a PID control in the maintaining phase can also be adopted to further ensure the desired current output (see Fig. 2b). Note that the PID control acting in the maintaining phase will not affect the response time in the lifting phase. Simply, the next cycle or a current sequence is able to be achieved by repeating the above process. In simple terms, the synergistic strategy of SSBC current source is to control the switching time of supercapacitor for a fast lifting phase followed by the adjustment of PWM duty cycle of Buck converter for a maintaining phase. According to this, the SSBC current source is programmable to produce a desired current waveform or a current sequence (see Fig. 2c). Correspondingly, a programmable output of a sequence of magnetic field strength in MRD can be realized. Another phenomenon in Fig. 2c is that the current can be rapidly loaded in the lifting process to reach a higher level, but how to rapidly reach a lower level since the current in the magnetic coil needs time to dissipate. One method is that we can add a diode in parallel with the coil. The diode is not on when the coil is applied by a current; when the coil in unloaded, it forms a reverse electrodynamic potential, so that the diode is on and forms a discharge loop for rapid discharge. Such a simple strategy manifests that the SSBC current source would be economical and affordable for commercial applications of MRD. Also, it should not only work for MRD but also for other MR devices as a result of their essentially similar electrical properties.

**Figure 3:**
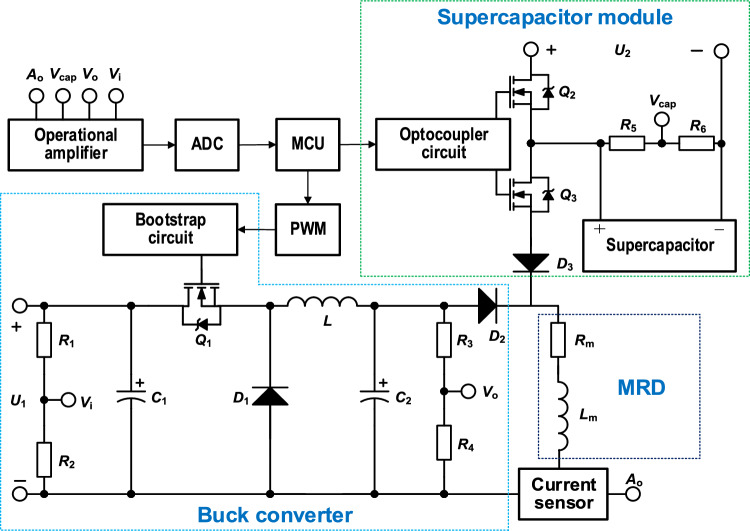
Schematic of the proposed SSBC current source for MRD based on synergy between supercapacitor and Buck converter.

Based on the design strategy above, Fig. 3 provides a detailed schematic of the proposed SSBC current source. In Fig. 3, the supercapacitor can be composed of multiple ones actually. *Q*_2_ and *Q*_3_ are NMOS-FET (n-channel metal–oxide–semiconductor field-effect transistor). The MCU controls *Q*_2_ and *Q*_3_ via an optocoupler driving circuit, and then the charge and discharge states of the supercapacitor are controlled. *C*_1_ denotes the input filter capacitor, *C*_2_ the output filter capacitor of Buck converter, *L* the filter inductor, *D*_1_ and* C*_2_ the freewheeling diodes, *Q*_1_ the switch NMOS-FET of Buck converter, *D*_3_ and *D*_4_ the diodes preventing current backflow, *V*_i_ and *V*_o_ the sampling input voltage and output voltage of Buck converter, *V*_cap_ the voltage of supercapacitor, and *A*_o_ the final output current for MRD.

## Theoretical modeling of circuits

The SSBC current source comprises a lifting phase contributed by supercapacitor and a following maintaining phase contributed by Buck converter. Here, we establish circuit models with supercapacitor directly as a current source (i.e., SD current source) for MRD and with Buck converter directly as a current source (i.e., BCD current source) for MRD, respectively. Then, we can compare and analyze their response time and provide a base for the design of a practical SSBC current source.

### Modeling of SD current source for MRD

**Figure 4:**
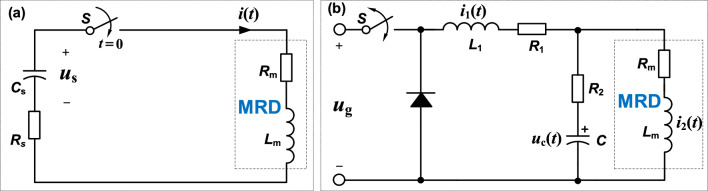
(**a**) Simple circuit model including supercapacitor directly as current source (i.e., SD current source) for MRD, and (**b**) circuit model including Buck converter directly (considering the filter inductance and parasitic capacitance) as current source (i.e., BCD current source) for MRD.

The equivalent circuit of SD current source is shown in Fig. 4a. It can be simplified as an *RLC* series circuit with zero input response. *C*_s_, *R*_s_, and *u*_s_ are the capacitance, resistance, and initial voltage of supercapacitor, respectively. *S* denotes the switch (NMOS-FET). At *t* = 0, switch *S* is turned on, *u*_s_ (0_-_) = *u*_0_, *i*_*t*_ (0_-_) = 0, and the supercapacitor begins to discharge, *u*_s_ (0_+_) = *u*_s_ (0_-_) = *u*_0_, *i*_*t*_ (0_+_) = *i*_*t*_ (0_-_) = 0.

According to the Kirchhoff’s voltage law, we know

2 $$L_{{\text{m}}} C_{{\varvec{s}}} \frac{{d^{2} u_{{\varvec{s}}} (t)}}{{dt^{2} }} + \left( {R_{{\text{m}}} + R_{{\varvec{s}}} } \right)C_{{\varvec{s}}} \frac{{du_{{\varvec{s}}} (t)}}{dt} + u_{{\varvec{s}}} (t) = 0$$

In an actual circuit, because *R*_m_ + *R*_s_ > 2 $\sqrt{{\text{L}}_{\rm{m}}/{\text{C}}_{\rm{s}}}$, we only discuss the overdamping case. Then, the voltage response of the SD current source is

3 $$u_{{\mathbf{s}}} (t) = \frac{{U_{0} }}{{p_{2} - p_{1} }}\left( {p_{2} e^{{p_{1} t}} - p_{1} e^{{p_{2} t}} } \right)$$

Thus, the current response on the MRD load is

4 $$i(t) = C_{\mathbf{s}} \frac{{du_{\mathbf{s}} (t)}}{{dt}} = \frac{{U_{0} }}{{L_{{\text{m}}} \left( {p_{2} - p_{1} } \right)}}\left( {e^{{p_{1} t}} - e^{{p_{2} t}} } \right)$$

where

5 $$\left\{ \begin{gathered} p_{1} = - \frac{{R_{{\rm{m}}} + R_{{\varvec{s}}} }}{{2L_{{\rm{m}}} }} + \sqrt {\left( {\frac{{R_{{\rm{m}}} + R_{{\varvec{s}}} }}{{2L_{{\rm{m}}} }}} \right)^{{2}} - \frac{1}{{L_{{\rm{m}}} C_{{\varvec{s}}} }}} \hfill \\ p_{2} = - \frac{{R_{{\rm{m}}} + R_{{\varvec{s}}} }}{{2L_{{\rm{m}}} }} - \sqrt {\left( {\frac{{R_{{\rm{m}}} + R_{{\varvec{s}}} }}{{2L_{{\rm{m}}} }}} \right)^{{2}} - \frac{1}{{L_{{\rm{m}}} C_{{\varvec{s}}} }}} \hfill \\ \end{gathered} \right.$$

### Modeling of BCD current source for MRD

Figure 4b shows the equivalent circuit of the BCD current source. We employ the state-space averaging principle for the modeling. Here, the equivalent series resistance* R*_1_ of filter inductor *L*_1_ and the equivalent filter resistance* R*_2_ of filter capacitor *C* are considered, so that the theoretical model fits into the actual situation more. The principle is descripted as follows. The state variables are the current of filter inductor* L*_1_ and the voltage of filter capacitor *C*. The state equation is given according to the on and off states of switch *S*. Then, the average state variables are given by means of the time averaging method and, further, the state equations during the entire switching cycle are obtained. At last, based on the assumption of low frequency and small ripple, the model is performed by a separation perturbation. By ignoring the high-order components, a simplified linear circuit is obtained, and finally a small signal model for the BCD current source is established.

Thus, the state space equation of BCD current source is given as

6 $$\left\{ \begin{gathered} \mathop {\varvec{X}}\limits^{ \bullet } { + }\mathop {\hat{\user2{x}}}\limits^{ \bullet } (t){ = }{\varvec{AX}} + {\varvec{BU}} + \user2{A\hat{x}}(t) + \user2{B\hat{u}}(t) + \left[ {\left( {{\varvec{A}}_{1} - {\varvec{A}}_{2} } \right){\varvec{X}} + \left( {{\varvec{B}}_{1} - {\varvec{B}}_{2} } \right){\varvec{U}}} \right]\hat{d}(t) \hfill \\ {\kern 1pt} {\kern 1pt} {\kern 1pt} {\kern 1pt} {\kern 1pt} {\kern 1pt} {\kern 1pt} {\kern 1pt} {\kern 1pt} {\kern 1pt} {\kern 1pt} {\kern 1pt} {\kern 1pt} {\kern 1pt} {\kern 1pt} {\kern 1pt} {\kern 1pt} {\kern 1pt} {\kern 1pt} {\kern 1pt} {\kern 1pt} {\kern 1pt} {\kern 1pt} {\kern 1pt} {\kern 1pt} {\kern 1pt} {\kern 1pt} {\kern 1pt} {\kern 1pt} {\kern 1pt} {\kern 1pt} {\kern 1pt} {\kern 1pt} {\kern 1pt} {\kern 1pt} {\kern 1pt} {\kern 1pt} {\kern 1pt} {\kern 1pt} {\kern 1pt} + \left( {{\varvec{A}}_{1} - {\varvec{A}}_{2} } \right)\hat{\user2{x}}(t)\hat{d}(t) + \left( {{\varvec{B}}_{1} - {\varvec{B}}_{2} } \right)\hat{\user2{u}}(t)\hat{d}(t) \hfill \\ {\varvec{Y}} + \hat{\user2{y}}(t) = {\varvec{CX}} + {\varvec{EU}} + \user2{C\hat{x}}(t) + \user2{E\hat{u}}(t) + \left[ {\left( {{\varvec{C}}_{1} - {\varvec{C}}_{2} } \right){\varvec{X}} + \left( {{\varvec{E}}_{1} - {\varvec{E}}_{2} } \right){\varvec{U}}} \right]\hat{d}(t) \hfill \\ {\kern 1pt} {\kern 1pt} {\kern 1pt} {\kern 1pt} {\kern 1pt} {\kern 1pt} {\kern 1pt} {\kern 1pt} {\kern 1pt} {\kern 1pt} {\kern 1pt} {\kern 1pt} {\kern 1pt} {\kern 1pt} {\kern 1pt} {\kern 1pt} {\kern 1pt} {\kern 1pt} {\kern 1pt} {\kern 1pt} {\kern 1pt} {\kern 1pt} {\kern 1pt} {\kern 1pt} {\kern 1pt} {\kern 1pt} {\kern 1pt} {\kern 1pt} {\kern 1pt} {\kern 1pt} {\kern 1pt} {\kern 1pt} {\kern 1pt} {\kern 1pt} {\kern 1pt} {\kern 1pt} {\kern 1pt} {\kern 1pt} {\kern 1pt} {\kern 1pt} + \left( {{\varvec{C}}_{1} - {\varvec{C}}_{2} } \right)\hat{\user2{x}}(t)\hat{d}(t) + \left( {{\varvec{E}}_{1} - {\varvec{E}}_{2} } \right)\hat{\user2{u}}(t)\hat{d}(t) \hfill \\ \end{gathered} \right.$$

where

7 $$\left\{ \begin{gathered} {\varvec{A}} = D_{1} {\varvec{A}}_{1} + D_{2} {\varvec{A}}_{2} \hfill \\ {\varvec{B}} = D_{1} {\varvec{B}}_{1} + D_{2} {\varvec{B}}_{2} \hfill \\ {\varvec{C}} = D_{1} {\varvec{C}}_{1} + D_{2} {\varvec{C}}_{2} \hfill \\ {\varvec{E}} = D_{1} {\varvec{E}}_{1} + D_{2} {\varvec{E}}_{2} \hfill \\ \end{gathered} \right.$$

***X***, ***U***, ***Y*** are the DC components of the state vector, input vector, and output vector, respectively. $\widehat{\text{x}}({\text{t}})$, $\widehat{{\varvec{u}}}({\text{t}})$, $\widehat{\text{y}}({\text{t}})$ are the corresponding small AC signal components. ***A***_1_, ***B***_1_, ***C***_1_, ***E***_1_ are the state matrix, input matrix, output matrix, and transfer matrix at the on states of switch *S*, respectively. ***A***_2_, ***B***_2_, ***C***_2_, ***E***_2_ are the related matrixes at the off states. *D*_1_ and *D*_2_ denote the DC components of duty cycle, while $\widehat{\text{d}}({\text{t}})$ denotes the small signal components of duty cycle.

In Eq. (6), the DC components on both sides are correspondingly equal, and the DC component ***X*** is constant. Then, we get the quiescent operating point of the BCD current source as

8 $$\left\{ \begin{gathered} {\varvec{X}} = - {\varvec{A}}^{ - 1} {\varvec{BU}} \hfill \\ {\varvec{Y}} = \left( {{\varvec{E}} - {\varvec{CA}}^{ - 1} {\varvec{B}}} \right){\varvec{U}} \hfill \\ \end{gathered} \right.$$

Also, in Eq. (6), the small AC signal components on both sides are correspondingly equal, and the amplitude of small AC signals is much smaller than that of the DC components. Ignoring the high-order small signal components, the equation is linearized to obtain the state equation and output equation of small AC signal, as follows:

9 $$\left\{ \begin{gathered} \mathop {\widehat{{\varvec{x}}}}\limits^{ \bullet } (t) = \user2{A\hat{x}}(t) + \user2{B\hat{u}}(t) + \left[ {\left( {{\varvec{A}}_{1} - {\varvec{A}}_{2} } \right){\varvec{X}} + \left( {{\varvec{B}}_{1} - {\varvec{B}}_{2} } \right){\varvec{U}}} \right]\hat{d}(t) \hfill \\ \hat{\user2{y}}(t) = \user2{C\hat{x}}(t) + \user2{E\hat{u}}(t) + \left[ {\left( {{\varvec{C}}_{1} - {\varvec{C}}_{2} } \right){\varvec{X}} + \left( {{\varvec{E}}_{1} - {\varvec{E}}_{2} } \right){\varvec{U}}} \right]\hat{d}(t) \hfill \\ \end{gathered} \right.$$

Assuming that the initial values of state variables are zero, applying Laplace transform to Eq. (9) yields the small signal model in *s* domain as

10 $$\left\{ {\begin{array}{*{20}l} {s{{\hat{\mathbf{x}}}}(s) = {{\mathbf{{A}}{\hat{\mathbf{x}}}}}(s) + {{\mathbf{{B}}{\hat{\mathbf{u}}}}}(s) + \left[ {\left( {{{\mathbf{A}}}_{1} - {{\mathbf{A}}}_{2} } \right){{\mathbf{X}}} + \left( {{{\mathbf{B}}}_{1} - {{\mathbf{B}}}_{2} } \right){{\mathbf{U}}}} \right]\hat{d}(s)} \hfill \\ {{\hat{{\mathbf{y}}}}(s) = {{\mathbf{{C}}{\hat{\mathbf{x}}}}}(s) + \left( {{{\mathbf{C}}}_{1} - {{\mathbf{C}}}_{2} } \right){{\mathbf{X}}}\hat{d}(s)} \hfill \\ \end{array} } \right.$$

Hence, the open loop transfer function *G* (*s*) of BCD current source is derived when the input voltage perturbation is zero, as follows:

11 $$G(s) = \;\left. {\frac{{\widehat{{\mathbf{x}}}(s)}}{{\widehat{{\mathbf{d}}}(s)}}} \right|_{{{\hat{\mathbf{u}}}(s) = 0}} = (s{\mathbf{I}} - {\mathbf{A}})^{ - 1} \;\left[ {\left( {{\mathbf{A}}_{1} - {\mathbf{A}}_{2} } \right){\mathbf{X}} + \left( {{\mathbf{B}}_{1} - {\mathbf{B}}_{2} } \right){\mathbf{U}}} \right]$$

At this point, the dynamic response of BCD current source can be analyzed based on this transfer function in Eq. (11). A further step is to obtain the solution for the BCD current source with the MRD as a load.

In Fig. 4b, *i*_1_ (*t*) and *i*_2_ (*t*) are the currents of filter inductor* L*_1_ and equivalent inductor *L*_m_ of MRD, respectively. *u*_g_ (*t*) and *u*_c_ (*t*) are the input voltage and the voltage of filter capacitor *C*, respectively. *i*_o_ (*t*) (i.e., = *i*_2_ (*t*)) and *u*_o_ (*t*) are the output current and output voltage on MRD as the load (i.e., *L*_m_ and *R*_m_ in cascade). We define the state vector x(t) = [i1 (t), uc (t), i2 (t)]^T^, input vector ***u***(t) = [ug (t)]^T^, and output vector ***y***(t) = [io (t), uo (t)]^T^. *T*_s_ is the PWM period.

When 0 < *t* < *dT*_s_, switch *S* is on. The state equation is

12 $$\begin{gathered} \left[ \begin{gathered} {i}_{1} (t) \hfill \\ {u}_{{\text{c}}} (t) \hfill \\ {i}_{2} (t) \hfill \\ \end{gathered} \right] = \left[ {\begin{array}{*{20}c} {\frac{{ - \left( {R_{1} + R_{2} } \right)}}{{L_{1} }}} & { - \frac{1}{{L_{1} }}} & {\frac{{R_{2} }}{{L_{1} }}} \\ {\frac{1}{C}} & 0 & { - \frac{1}{C}} \\ {\frac{{R_{2} }}{{L_{{\text{m}}} }}} & {\frac{1}{{L_{{\text{m}}} }}} & {\frac{{ - \left( {R_{{\text{m}}} + R_{2} } \right)}}{{L_{{\text{m}}} }}} \\ \end{array} } \right]\left[ \begin{gathered} i_{1} (t) \hfill \\ u_{{\text{c}}} (t) \hfill \\ i_{2} (t) \hfill \\ \end{gathered} \right] + \left[ {\begin{array}{*{20}c} {\frac{1}{{L_{1} }}} \\ 0 \\ 0 \\ \end{array} } \right]\left[ {u_{{\text{g}}} (t)} \right] \hfill \\ \left[ \begin{gathered} i_{{\text{o}}} (t) \hfill \\ u_{{\text{o}}} (t) \hfill \\ \end{gathered} \right] = \left[ {\begin{array}{*{20}c} \begin{gathered} 0 \hfill \\ R_{2} \hfill \\ \end{gathered} & \begin{gathered} 0 \hfill \\ 1 \hfill \\ \end{gathered} & \begin{gathered} 1 \hfill \\ - R_{2} \hfill \\ \end{gathered} \\ \end{array} } \right]\left[ \begin{gathered} i_{1} (t) \hfill \\ u_{{\text{c}}} (t) \hfill \\ i_{2} (t) \hfill \\ \end{gathered} \right] + \left[ \begin{gathered} 0 \hfill \\ 0 \hfill \\ \end{gathered} \right]\left[ {u_{{\text{g}}} (t)} \right] \hfill \\ \end{gathered}$$

where

13 $$\left\{ \begin{gathered} {\mathbf{A}}_{1} = \left[ {\begin{array}{*{20}c} {\frac{{ - \left( {R_{1} + R_{2} } \right)}}{{L_{1} }}} & { - \frac{1}{{L_{1} }}} & {\frac{{R_{2} }}{{L_{1} }}} \\ \frac{1}{C} & 0 & { - \frac{1}{C}} \\ {\frac{{R_{2} }}{{L_{{\text{m}}} }}} & {\frac{1}{{L_{{\text{m}}} }}} & {\frac{{ - \left( {R_{{\text{m}}} + R_{2} } \right)}}{{L_{{\text{m}}} }}} \\ \end{array} } \right], \hfill \\ {\mathbf{B}}_{1} = \left[ {\begin{array}{*{20}c} {\frac{1}{{L_{1} }}} \\ 0 \\ 0 \\ \end{array} } \right], \, {\mathbf{C}}_{1} = \left[ {\begin{array}{*{20}c} \begin{gathered} 0 \hfill \\ R_{2} \hfill \\ \end{gathered} & \begin{gathered} 0 \hfill \\ 1 \hfill \\ \end{gathered} & \begin{gathered} 1 \hfill \\ - R_{2} \hfill \\ \end{gathered} \\ \end{array} } \right], \, {\mathbf{E}}_{1} = \left[ \begin{gathered} 0 \hfill \\ 0 \hfill \\ \end{gathered} \right] \hfill \\ \end{gathered} \right.$$

When *dT*_s_ < *t* < *T*_s_, switch *S* is off. The state equation is

14 $$\begin{gathered} \left[ \begin{gathered} \mathop i\limits_{1} (t) \hfill \\ \mathop u\limits_{{\text{c}}} (t) \hfill \\ \mathop i\limits_{2} (t) \hfill \\ \end{gathered} \right] = \left[ {\begin{array}{*{20}c} {\frac{{ - \left( {R_{1} + R_{2} } \right)}}{{L_{1} }}} & { - \frac{1}{{L_{1} }}} & {\frac{{R_{2} }}{{L_{1} }}} \\ \frac{1}{C} & 0 & { - \frac{1}{C}} \\ {\frac{{R_{2} }}{{L_{{\text{m}}} }}} & {\frac{1}{{L_{{\text{m}}} }}} & {\frac{{ - \left( {R_{{\text{m}}} + R_{2} } \right)}}{{L_{{\text{m}}} }}} \\ \end{array} } \right]\left[ \begin{gathered} i_{1} (t) \hfill \\ u_{{\text{c}}} (t) \hfill \\ i_{2} (t) \hfill \\ \end{gathered} \right] + \left[ {\begin{array}{*{20}c} 0 \\ 0 \\ 0 \\ \end{array} } \right]\left[ {u_{{\text{g}}} (t)} \right] \hfill \\ \left[ \begin{gathered} i_{{\text{o}}} (t) \hfill \\ u_{{\text{o}}} (t) \hfill \\ \end{gathered} \right] = \left[ {\begin{array}{*{20}c} \begin{gathered} 0 \hfill \\ R_{2} \hfill \\ \end{gathered} & \begin{gathered} 0 \hfill \\ 1 \hfill \\ \end{gathered} & \begin{gathered} 1 \hfill \\ - R_{2} \hfill \\ \end{gathered} \\ \end{array} } \right]\left[ \begin{gathered} i_{1} (t) \hfill \\ u_{{\text{c}}} (t) \hfill \\ i_{2} (t) \hfill \\ \end{gathered} \right] + \left[ \begin{gathered} 0 \hfill \\ 0 \hfill \\ \end{gathered} \right]\left[ {u_{{\text{g}}} (t)} \right] \hfill \\ \end{gathered}$$

where

15 $$\left\{ \begin{gathered} {\mathbf{A}}_{2} = \left[ {\begin{array}{*{20}c} {\frac{{ - \left( {R_{1} + R_{2} } \right)}}{{L_{1} }}} & { - \frac{1}{{L_{1} }}} & {\frac{{R_{2} }}{{L_{1} }}} \\ \frac{1}{C} & 0 & { - \frac{1}{C}} \\ {\frac{{R_{2} }}{{L_{{\text{m}}} }}} & {\frac{1}{{L_{{\text{m}}} }}} & {\frac{{ - \left( {R_{{\text{m}}} + R_{2} } \right)}}{{L_{{\text{m}}} }}} \\ \end{array} } \right], \hfill \\ {\mathbf{B}}_{2} = \left[ {\begin{array}{*{20}c} 0 \\ 0 \\ 0 \\ \end{array} } \right],{\mathbf{C}}_{2} = \left[ {\begin{array}{*{20}c} \begin{gathered} 0 \hfill \\ R_{2} \hfill \\ \end{gathered} & \begin{gathered} 0 \hfill \\ 1 \hfill \\ \end{gathered} & \begin{gathered} 1 \hfill \\ - R_{2} \hfill \\ \end{gathered} \\ \end{array} } \right],{\mathbf{E}}_{2} = \left[ \begin{gathered} 0 \hfill \\ 0 \hfill \\ \end{gathered} \right] \hfill \\ \end{gathered} \right.$$

Substituting Eqs. (13) and (15) into (11) yields the transfer function* H* (*s*) from duty cycle to output current as

16 $$H(s) = \frac{{\left( {1 + R_{2} Cs} \right)U_{g} }}{{CL_{1} L_{{\text{m}}} s^{3} + \left[ {\left( {R_{1} + R_{2} } \right)CL_{{\text{m}}} + \left( {R_{{\text{m}}} + R_{2} } \right)CL_{1} } \right]s^{2} + \left( {L_{1} + L_{{\text{m}}} + R_{1} R_{{\text{m}}} C + R_{{\text{m}}} R_{2} C + R_{1} R_{2} C} \right)s + R_{1} + R_{{\text{m}}} }}$$

## Theoretical results and discussion

Here, we visualize the theoretical results, i.e., current responses of SD and BCD current sources according to the mathematical models established above. Further, the current response of SSBC current source is simulated along with the magnetic induction in the MRD. The parameters of coil resistance* R*_m_ and inductance *L*_m_ are from an actual MRD, i.e., *R*_m_ = 1.3 Ω, *L*_m_ = 25 mH.

### Simulated response of SD current source for MRD

**Figure 5:**
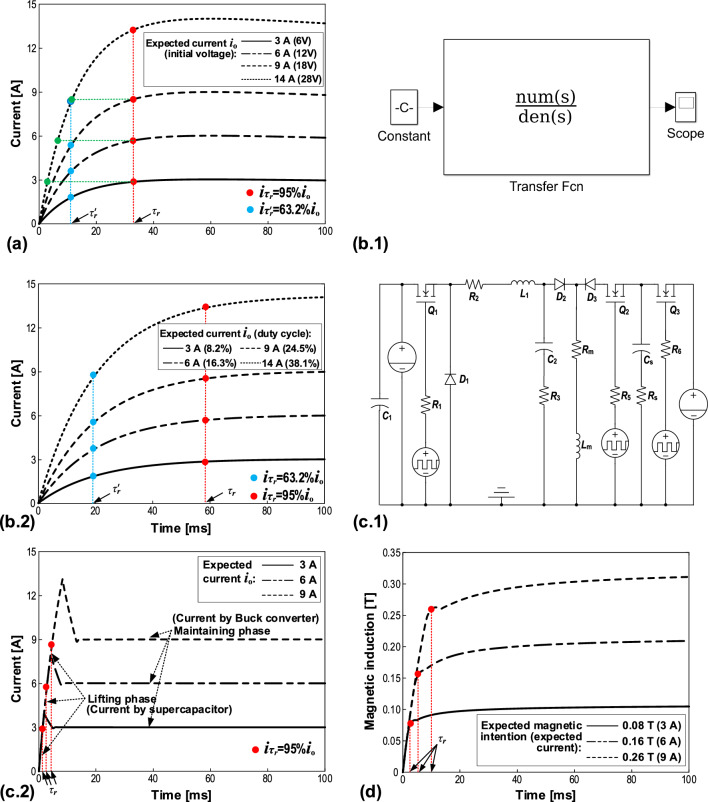
Simulations. (**a**) Current responses of SD current source with various initial voltages of supercapacitors. (**b**): (**b.1**) MATLAB/Simulink simulation of the BCD current source, and (**b.2**) the related current responses with various PWM duty cycles. (**c**): (**c.1**) Saber simulation of the proposed SSBC current source, and (**c.2**) its current responses with various expected values. (**d**) Simulated responses of magnetic field in the damping channel of MRD (without MR fluid) related to the currents in (**c.2**) produced by SSBC current source. Note that there are overshoots in the current responses in (**c.2**) but they almost disappear in the magnetic induction curves in (**d**). All the simulated results are obtained based on the actual MRD as a load. The red and blue points in (**a**), (**b.2**), and (**c.2**) denote the time when the currents reach 95% (${\tau }_{r}$) and 63.2% (${\tau }_{r}{\prime}$) of the expected outputs, respectively. The red points in (**d**) denote the turning points. The green points in (**a**) mark the values on the 14 A curve which match those (red points) on the curves of 3, 6 and 9 A, correspondingly.

Based on Fig. 4a and Eq. (4), Fig. 5a shows the simulated current response of SD current source. We can know the response time from the current curves. Generally, response time is defined as the time that current reaches 95% or 63.2% of its steady value (i.e., expected value of current), which is denoted as ${\tau }_{r}$ and ${\tau }_{r}{\prime}$, respectively. We will give both types of response time, but ${\tau }_{r}$ is selected for specified comparisons since it exists in most previous studies.

The current curves in Fig. 5a are simulated when the initial voltages of the supercapacitor are 6.0, 11.9, 17.8, and 27.7 V, respectively. The corresponding expected currents are 3, 6, 9, and 14 A, respectively; these large currents should fulfill the requirements in most applications. It is worth mentioning that the resistance of supercapacitor is 0.6 Ω, which is close to the coil resistance (1.3 Ω) of the MRD and thus cannot be ignored. Regarding the response time, all curves exhibit ${\tau }_{r}{\prime}=$ 12.2 ms (i.e., @ 63.2% of expected value, marked by blue points) and ${\tau }_{r}=$ 33.3 ms (i.e., @ 95% of expected value, marked by red points). This property is determined by Eq. (4). Such long response time values ​​are much longer than that of the material of MR fluid, indicating that the SD current source is not competent for the real-time control of MRD. There is a slightly declining trend over time on the 14 A curve after it reaches the expected value. This is because the supercapacitor discharges such a large current relatively fast due to its low energy density. In fact, this issue can be readily solved by a recharging strategy using multiple supercapacitors. Also, as predicted, the expected value of current is positively related to the initial voltage of the supercapacitor.

### Simulated response of BCD current source for MRD

Similarly, according to Fig. 4b and Eq. (16), the simulated current response of the BCD current source is obtained, as shown in Fig. 5b.2. Figure 5b.1 illustrates the MATLAB / Simulink simulation with a system block diagram based on the transfer function in Eq. (16). In the simulation, input is the duty cycle of PWM signal, while output is the current on the MRD. To compare with the results of SD current source in Fig. 5a, the duty cycles are set to ​8.2%, 16.3%, 24.5% and 38.1% so as to produce the same expected currents of 3, 6, 9 and 14 A, respectively. Apparently, the expected current is approximately linear to the duty cycle. As for the response time, all curves present ${\tau }_{r}{\prime}=$ 18.9 ms (marked by blue points) and ${\tau }_{r}=$ 58.1 ms (marked by red points) in Fig. 5b.2, which are 1.55 times and 1.74 times of those of the SD current source, respectively. Clearly, the BCD current source responds slower than the SD current source, which indicates the advantage of supercapacitor. However, it is known that both SD and BCD current sources do not qualify for the real-time control of MRD.

### Simulated response of SSBC current source for MRD

The induced current caused by the coil inductance is a major cause for the slow response of SD and BCD current sources. Let us take a closer observation at Fig. 5a of the SD current source. First, the larger the initial voltage of supercapacitor, the higher the expected current, and the faster the current rises. Second, focusing on the 14 A curve (i.e., the largest expected current), we can see that it grows fastest (because all curves have the same response time ${\tau }_{r}$ or ${\tau }_{r}{\prime}$). Although it still takes ${\tau }_{r}=$ 33.3 ms to reach 95% of 14 A, it spends much less time in passing the expected values of the other 3 curves (marked by green points in Fig. 5a). Based on this truth as well as the design strategy described in Sect. 1.2 for SSBC current source, we first use a supercapacitor with a higher initial voltage to rapidly lift the current. And then, like a “relay race”, the Buck converter maintains the output of the expected current. Namely, we call this strategy the synergy between supercapacitor contributing to the lifting phase and Buck converter contributing to the maintaining phase. Note that, to achieve a faster rise of magnetic induction, it is necessary to produce a current overshoot before the Buck converter acts. This overshoot could help to reduce the effect caused by the coil and eddy current.

According to Figs. 2b and 3, we employ the Saber software for the simulation of SSBC current source, with a model shown in Fig. 5c.1. Figure 5c.2 presents the simulated results for the expected currents of 3, 6 and 9 A. In the lifting phases, an allowable high voltage (44 V) of the supercapacitor was used for all cases to obtain the responses as fast as possible. According to Fig. 5c.2, the response times ${\tau }_{r}$ (i.e., @ 95% of expected value) are 1.3, 2.7 and 4.5 ms for 3, 6 and 9 A curves, respectively. These values show that the SSBC current source is up to 24.6, 11.3 and 6.4 times faster than the SD current source (Fig. 5a), and up to 43.7, 21.5 and 12.9 times faster than the BCD current source (Fig. 5b.2), respectively. Surprisingly, the SSBC current source extraordinarily speeds up the current output for MRD, thanks to the synergy between supercapacitor and Buck converter.

### Simulated response of magnetic field in MRD by SSBC current source

The excitation coil is the only electrical part in MRD. The magnetic field generated by the coil is the direct excitation of the control medium of MR fluid. In other words, the response time of magnetic field is essentially the key to the fast control of rheology of the MR fluid in MRD. Therefore, we should pay attention to the response time of not only the current but also the magnetic field. Here, the Ansoft software is used to simulate the magnetic field response. The simulation is based on the actual structure, materials and coil windings of the MRD (without MR fluid). The input is the current data of SSBC current source in Fig. 5c.2, while the output is the magnetic induction in the damping channel of MRD. The eddy current of iron core is also considered in the simulation.

Figure 5d exhibits the simulated results. It can be seen that the magnetic induction of each curve first rises to a turning point rapidly, and then tends to level off. We label each curve with the value at their turning points, and the time at 95% of which is defined as the response time. In this way, the magnetic inductions of the current of 3, 6 and 9 A are 0.08, 0.16 and 0.26 T, and the response times are 2.5, 5 and 8 ms, respectively. The data indicate that the magnetic induction is approximately proportional to the current and, hence, proportional to the duty cycle of PWM signal. This property means a simple control operation in applications. Besides, there exists a delay between the magnetic inductions and currents, specifically 1.2 ~ 3.5 ms. This delay shows that factors such as eddy current are of significance for the response of magnetic field. In addition, the magnetic inductions are not very great because there is no MR fluid simulated in the damping channel. Interestingly, unlike the overshoots of current existing in Fig. 5c.2, the overshoot of magnetic induction almost disappears in Fig. 5d. This is likely due to the significant eddy current effect in the piston head of MRD. Specifically, during the rapid rise of current in the lifting phase, the overshoot is offset by a converse magnetic field generated by the eddy current. The induced magnetic field of induced coil current plays a similar role. It suggests that there would be no impact of the current overshoot of SSBC current source on the damping force either. The overshoots would be endurable because they last for as short as only a couple of ms. It means that the overshoots produce very limited power in the coil and the little heat consumption would not be harmful to the coil of the MRD. Also, other electrical components can be selected to endure the overshoots.

## Experimental results and discussion

**Figure 6:**
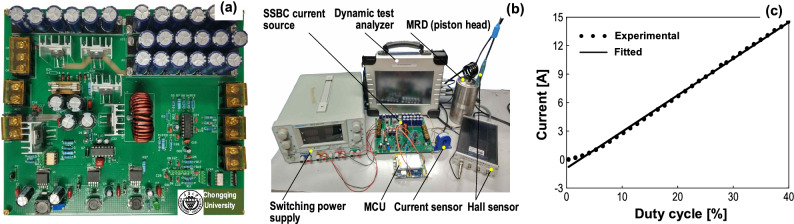
(**a**) Prototype of the SSBC current source for MRD, (**b**) experimental setup of testing system, and (**c**) relationship between output current and PWM duty cycle of the Buck converter.

We designed and fabricated a prototype of the SSBC current source together with an experimental testing system, as shown in Fig. 6a and b, in order for verification of the proposed design strategy. In the simulations we know that the response with a lifting phase of supercapacitor (i.e., SSBC current source) is much faster than the cases without it (i.e., SD and BCD current sources). Here, we further investigated the lifting phase contributed by a Buck converter experimentally, i.e., a so-called SBCBC current source with buck converter for both the lifting phase and maintaining phase. In addition, the programmable SSBC current source was used to produce a sequence of expected currents along with a sequence of expected magnetic inductions. At last, the stability of response time of the SSBC current source was verified by 100 cycles of on/off repeatability tests.

In the experiments, a current sensor (LHB-D-Y2, Xinghui Electronics Co., Ltd., China) and a Tesla meter (CH-1300, Beijing Cuihaijiacheng Magnetic Technology Co., Ltd., China) were used, and the data were recorded with a dynamic analyzer (TPP-1600-17,001, Sichuan Top Measurement & Control Co., Ltd., China) at a sampling frequency of 10 kHz. The MCU (STM32F103) was used to control the PWM signal of Buck converter. The measured current of the Buck converter as a function of duty cycle is shown in Fig. 6c. It approximately exhibits a linear relationship between the current and duty cycle, and the fitted line is

17 $$i = 0.38D - 0.77$$

where *D* is the duty cycle and *i* is the current. Then, the output current could be readily adjusted by the duty cycle in the maintaining phase. A PID control was employed in the maintaining phase to stabilize the expected current influences such as the heating of MRD coil.

### Experimental responses of SD and BCD current sources for MRD

**Figure 7:**
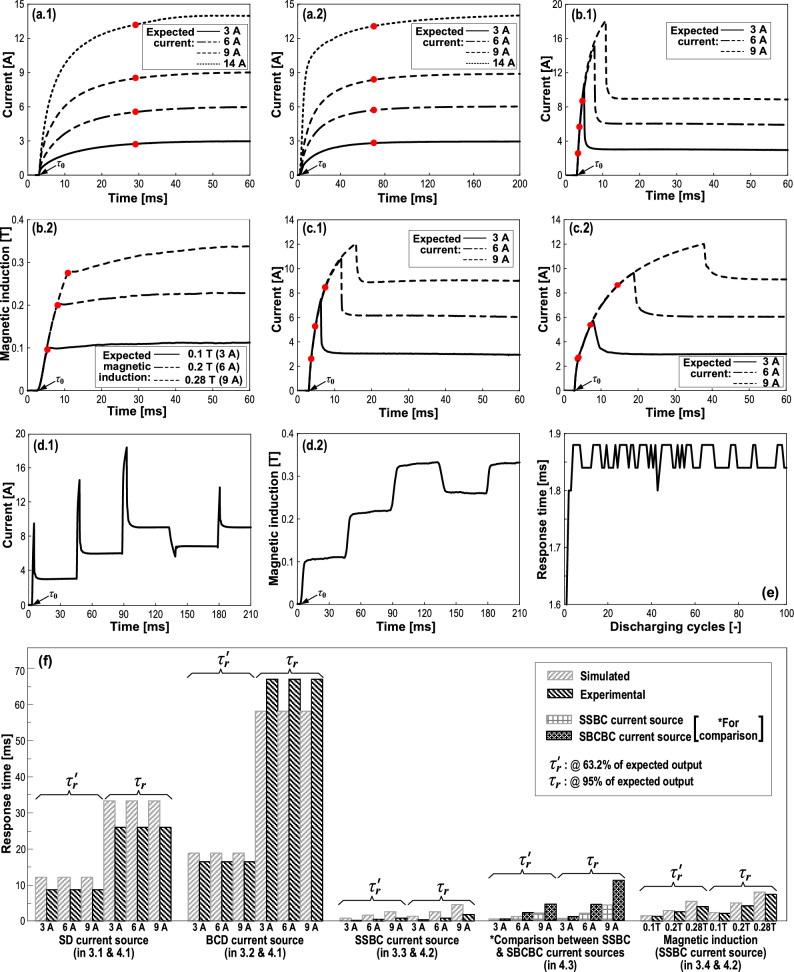
Experimental results. (**a**) Current responses of (**a.1**) the SD current source and (**a.2**) the BCD current source for MRD. (**b**) (**b.1**) Current response of the SSBC current source and (**b.2**) the related responses of magnetic field in the damping channel of MRD (without MR fluid). (**c**) Comparison between current responses of (**c.1**) SSBC current source with lifting phase by supercapacitor and (**c.2**) SBCBC current source with lifting phase by Buck converter. The red points in (**a**), (**b**) and (**c**) denote the response time (${\tau }_{r}$). ${\tau }_{0}$ indicates the beginning time points. (**d**) Programmable output of SSBC current source: (**d.1**) a current sequence and (**d.2**) related magnetic induction sequence. Note that there are overshoots on the current plateaus in (**d.1**) but none on the magnetic induction in (**d.2**). (**e**) Stability of the response time of SSBC current source over 100 cycles of on/off operations. (**f**) Integration of response time for all cases in this study.

Corresponding to the simulated results in Figs. 5a, b.2 and 7a.1, a.2 give the experimental results of SD current source and BCD current source, respectively. Similarly, the expected currents are 3, 6, 9, and 14 A in experiments. In general, the experimental curves have similar behaviors with the simulated ones. In Fig. 7a.1 and a.2, all curves measured have the same response time ${\tau }_{r}=$ 26 ms for the SD current source and ${\tau }_{r}=$ 67 ms for the BCD current source. Recalling the simulations of Fig. 5a and b.2, the former ${\tau }_{r}=$ 33.3 ms and the latter ${\tau }_{r}=$ 58.1 ms. Figure 7f integrates the response time data of all cases in this study. The difference between the experiments and simulations should mainly generate from nonideal circuit parameters. But, an identical truth is that the supercapacitor responds much faster than the Buck converter. Again, the experiments demonstrate that it is hard to achieve a real-time control of the MRD by directly using the SD or BCD current sources.

### Experimental response of SSBC current source for MRD

Corresponding to the simulated results in Figs. 5c.2, 7b.1 gives the experimental results of SSBC current source. The same initial voltage (44 V) of the supercapacitor is used as in the simulations. The magnetic induction in the MRD damping channel was measured by the Tesla sensor, as shown in Fig. 7b.2. According to Fig. 7b.1, the measured response times ${\tau }_{r}$ ​​are as fast as 0.44, 0.84 and 1.88 ms for the 3, 6 and 9 A curves, respectively; these values can be highlighted as the fastest level compared to previous studies. Recalling Fig. 5c.2, the simulated ones ${\tau }_{r}$ ​​are 1.3, 2.7 and 4.5 ms, correspondingly. It shows that the experiments respond faster than the simulations, and the mismatch should also be caused mainly by nonideal circuit parameters.

Regarding the magnetic inductions (see Fig. 7b.2), the experimental response times are 2.2, 4.2 and 7.4 ms for the 0.1, 0.2 and 0.28 T curves, respectively. The response time rises as the magnetic induction increases. They are also slightly faster than the simulated results ​​(2.5, 5 and 8 ms). Figure 7f clearly shows the differences between all these response times. There is also an approximately linear relationship between the magnetic induction and current (and also the PWM duty cycle). Similarly, the overshoot disappears in the magnetic induction. Besides, the magnetic induction exhibits a delay to the current as well, specifically 1.8 ~ 5.5 ms. Nevertheless, a response time of 2.2 ~ 7.4 ms of magnetic field would satisfy many applications of MRD. In the cases that require faster response, efforts should be made to further improve the response speed such as eliminating the eddy current of iron core.

### Experimental comparison between responses with lifting phase by supercapacitor and by Buck converter

An interesting question is what is the difference between the effect of using a supercapacitor and a Buck converter in the lifting phase. The difference between the SSBC current source and SBCBC current source is that the former has a lifting phase by supercapacitor while the latter has one by Buck converter. The same point is that both have Buck converter for the maintaining phase. First, we set the same expected current of 14 A (i.e., the 14 A curves in Fig. 7a.1 and a.2) for the lifting phases of the two current sources, corresponding to an initial voltage of 27.3 V for the supercapacitor, and an input voltage of 48 V together with a duty cycle of 38.8% for the Buck converter. Then, the switching time was controlled to obtain the desired currents with overshoot in the lifting phases. The results are presented in Fig. 7c.1 and c.2 as well as in (f). It shows that the response time ${\tau }_{r}$ of the SSBC current source are 0.7, 2.2 and 4.3 ms for the 3, 6 and 9 A curves, and the related ${\tau }_{r}$ of the SBCBC current source are 1.2, 4.6 and 11.2 ms, respectively. It can be seen that the supercapacitor performs much better than the Buck converter in the lifting phases, which further confirms the superiority of supercapacitor. Nevertheless, the SBCBC current source responds much quicker than the SD and BCD current sources, indicating that applying a lifting phase can be an effective approach to accelerate the response for MRD.

### Programmable output of current / magnetic induction sequence of SSBC current source

All the simulations and experiments above have merely realized the output of single constant current. Most previous laboratory studies on MRDs were also performed under the excitation of constant current. This may be due to the lack of proper programmable current sources. However, a current source for MRD in practical applications must change its output rapidly to adjust to environmental requirements. Here, we employ the SSBC current source to continually produce an output of a sequence of expected currents / magnetic inductions. Figure 7d.1 gives a measured current sequence with five plateaus, i.e., 0 → 3 → 6 → 9 → 6.9 → 9 A. Figure 7d.2 shows the measured output of a sequence of the related magnetic inductions in the MRD damping gap, i.e., 0 → 0.1 → 0.2 → 0.28 → 0.23 → 0.28 T. It is worth pointing out that each plateau in the current sequence consists of a lifting phase and a maintaining phase. In the actual operations, we only need to programmatically control four primary variables: the order of lifting and maintaining phases, switching time of lifting phase, PWM duty cycle of Buck converter, and duration of the maintaining phase. This is very simple to realize programmatically. Therefore, the SSBC current source can be a high-speed, programmable and low-cost power supply for MRD and even other MR devices. It is believed to be promising in broad applications of MR technology.

### Stability of response time of SSBC current source

Supercapacitor is the key to ensure fast response of the SSBC current source. A repeatability test was performed in order to verify the stability of the response time of SSBC current source after periodic operations. The test was carried out with 100 cycles of on/off operations between 0 and 9 A. Each cycle was maintained for 100 ms with a standby time of 170 ms between neighboring cycles. The response time ${\tau }_{r}$ of each cycle was recorded. The supercapacitor was in charging state during the standby time to guarantee adequate electricity. As can be seen from Fig. 7e, the response time stabilizes between 1.84 and 1.88 ms with a fluctuation of only 1.1%. Such a high stability indicates a very reliable high-speed response of the SSBC current source.

## Conclusions

Current source is an indispensable component in MR systems for their applications. It is related to a key parameter, i.e., the response time of MR devices that determines the semi-active real-time controllability. In this study, we propose a programmable high-speed current source exclusively for MR devices (especially MRDs) based on the synergy between supercapacitor and Buck converter (i.e., SSBC current source).

The equivalent circuit model with an MRD as the load is presented. A challenge to design the current source for MRD is to overcome the effect of the induced current of inductive coil as well as the eddy current. The strategy of the SSBC current source features a lifting phase of supercapacitor followed by a maintaining phase of Buck converter. Specifically, the high power density of supercapacitor with a high initial voltage contributes to rapidly lifting the initial current with an overshoot. And then, like a “relay race”, the expected output current is maintained through a Buck converter. In simple terms, the synergistic strategy of SSBC current source is to control the switching time of supercapacitor for a fast lifting phase followed by adjustment of the PWM duty cycle of Buck converter for a maintaining phase. Besides, a PID control without affecting the response time is employed in the maintaining phase to protect the output from influences such as heating of the MRD coil.

Theoretical modelling and experiments are carried out for circuits incorporating supercapacitor directly as a current source (i.e., SD current source) for MRD and Buck converter directly as a current source (i.e., BCD current source) for MRD, as well as the SSBC current source. The output currents realized are 3, 6, and 9 A; these large currents should fulfill the requirements in most applications. Both simulations and experiments show that the BCD current source responds slower than the SD current source, which indicates the advantage of supercapacitor. Surprisingly, the SSBC current source extraordinarily speeds up the current output for MRD. In detail, its measured response times (@ 95% of expected output) ​​are 0.44, 0.84 and 1.88 ms for the 3, 6 and 9 A outputs, respectively; these values can be highlighted as the fastest level compared to previous studies. In addition, the simulated response for 3, 6 and 9 A outputs of the SSBC current source are up to 24.6, 11.3 and 6.4 times faster than the SD current source, and up to 43.7, 21.5 and 12.9 times faster than the BCD current source, respectively. This fact strongly proves the benefit from the synergy between supercapacitor and Buck converter, and indicates that it is hard to achieve a real-time control of the MRD by directly using the SD or BCD current sources.

The response time of magnetic field is essentially the key to the real-time control of rheology of the MR fluid in MRD. The results present that the magnetic induction is approximately proportional to the current as well as the duty cycle of PWM signal, which means a simple control operation in applications. The experimental response times are 2.2, 4.2 and 7.4 ms for the 0.1, 0.2 and 0.28 T outputs, respectively. Interestingly, the overshoot existing in the current almost disappears in the magnetic induction. Besides, the magnetic induction exhibits a delay of 1.2 ~ 3.5 ms to the current. Nevertheless, a response time of 2.2 ~ 7.4 ms of magnetic field would satisfy many applications of MRD. In the cases that require faster response, efforts should be made to further improve the response speed such as eliminating the eddy current of iron core.

We experimentally investigate the difference between the effect of using supercapacitor (i.e., SSBC current source) and Buck converter (i.e., SBCBC current source) in the lifting phase. It shows that the supercapacitor performs much better than the Buck converter in the lifting phases, which further confirms the superiority of supercapacitor. Nevertheless, the SBCBC current source responds much quicker than the SD and BCD current sources, indicating that applying a lifting phase can be an effective approach to accelerate the response for MRD.

The stability of response time of the SSBC current source is verified by 100 cycles of an on/off repeatability tests. The response time stabilizes with a fluctuation of only 1.1%; such a high stability indicates a very reliable high-speed response.

We employ the SSBC current source to produce a sequence of expected currents / magnetic inductions. Only four primary variables need to be controlled: the order of lifting and maintaining phases, switching time of lifting phase, PWM duty cycle of Buck converter, and duration of maintaining phase. Therefore, the SSBC current source can be a high-speed, programmable and low-cost power supply for MRD and even other MR devices in broad applications.

Future work will be on further improvement of the SSBC current source and the further delay for the damping force by the MRD under an external exciting movement with MR fluid filled in the damper.

The datasets used and/or analysed during the current study available from the corresponding author on reasonable request.
